# Quadricuspid Aortic Valve: Out of the Shadows, into the Light

**DOI:** 10.3390/diagnostics15131689

**Published:** 2025-07-02

**Authors:** Dmitri Panfilov, Elizaveta Petrakova, Boris Kozlov

**Affiliations:** Cardiology Research Institute, Branch of the Federal State Budgetary Scientific Institution ‘Tomsk National Research Medical Center of the Russian Academy of Sciences’, 634012 Tomsk, Russia

**Keywords:** quadricuspid aortic valve, echocardiography, aortic valve replacement

## Abstract

We present the case of a 63-year-old man with severe aortic valve regurgitation and left-ventricular dysfunction. The patient was scheduled for elective surgery. A quadricuspid aortic valve with fibrous thickening and calcification of the cusps was visualized intraoperatively while preoperative surface echocardiography had failed to diagnose this anomaly. The aortic valve was successfully replaced with a biological prosthesis.

**Figure 1:**
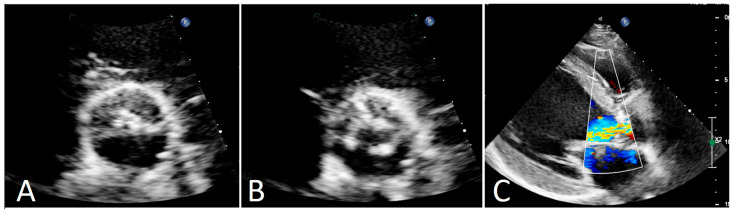
Congenital malformations of the aortic valve have different variants with various incidences in the population. Bicuspid aortic valves are the most common aortic anomaly (up to 2%), followed by unicuspid (0.02%) and then quadricuspid aortic valves (QAVs) (less than 0.01%) [1]. Historically, QAVs were encountered during open heart surgery or postmortem. Nowadays, in the majority of cases, the diagnosis is made by echocardiography (51%), intraoperatively (22.6%), and at autopsy (15.6%) [2]. To date, the literature regarding QAV characteristics is limited, with very little data describing disease presentation and treatment options. We present a rare case of surgical treatment of a patient with a QAV. A 63-year-old man complained of fatigue and moderate-to-severe dyspnea (NYHA class II) with a history of hypertension. Physical examination revealed diastolic murmur at the left-middle sternal border. Preoperative transthoracic echocardiography showed severe central aortic regurgitation as a result of malcoaptation of the presumably bicuspid aortic valve with concomitant mild-to-moderate aortic stenosis with a maximum gradient of 40 mm Hg (mean gradient of 22 mm Hg) and a valve area of 1.6 cm^2^ ((**A**)—systole, (**B**)—diastole, (**C**)—aortic regurgitation jet) (Figure 1). Left-ventricular dysfunction was present: end-diastolic diameter was 60 mm, ejection fraction was 40% with diffuse hypokinesis. Ascending aorta and aortic root were normal. Origins of the coronary arteries ostia were typical. Coronary angiography revealed no stenoses. The patient was scheduled to undergo elective surgery.

**Figure 2:**
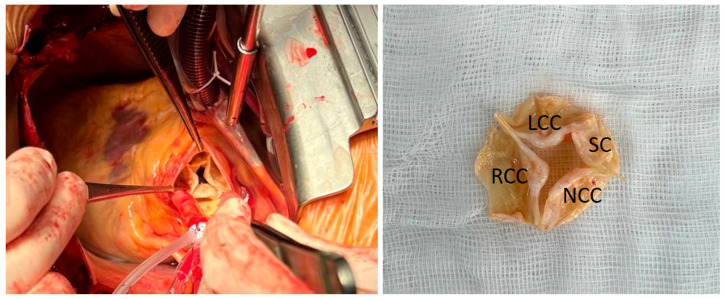
After cardiopulmonary bypass was established, the ascending aorta was cross clamped and transected. Selective cardioplegia in the left and right coronary ostia was given. During aortic root examination, the QAV was visualized and classified as type II by Nakamura classification (Figure 2). Due to partial calcification of the cusps and its malcoaptation caused by fibrous thickening, we did not attempt aortic valve repair. The aortic valve was excised (Excised aortic valve. LCC—left coronary cusp, RCC—right coronary cusp, NCC—noncoronary cusp, SC—supplementary cusp) (Figure 2) and replaced with a 23 mm biological prosthesis. Postoperative echocardiography showed no aortic regurgitation, and a mean gradient of 15 mm Hg. The patient had an uneventful postoperative course. Due to the extreme rarity of QAVs, precise preoperative assessment of the aortic valve plays a crucial role for surgical strategy. QAV is usually an isolated anomaly; however, in 18–32% of the patients it presents with a number of additional congenital heart defects including atrial and ventricular septal defects, sinus of Valsalva fistula, and subaortic fibromuscular stenosis [2]. The most frequent anomalies are coronary ostia malformations and displacement; they occur in 10% of such patients [1,3]. Awareness of these abnormalities is essential prior to the operation. Despite echocardiography being a leading modality for QAV detection, the echocardiographic findings do not always correlate with the surgical ones. Therefore, the value of the clinical features of the disease may be useful [4]. It is known that unlike patients with bicuspid aortic valve, patients with QAV are older irrespective of sex. Its most common clinical manifestation is aortic regurgitation (75%) or that combined with aortic stenosis (about 8%). Aortic stenosis alone may be present but is rare (about 1%) [2]. The vast majority of the QAV patients have a normal size ascending aorta and aortic root [5]. The surgical strategy for QAV includes aortic valve repair or replacement with biological/mechanical prosthesis, Ross procedure, Bentall procedure and even transcatheter aortic valve implantation [2,4]. Aortic valve repair is the preferable approach but the prognosis for cusp repair durability is undetermined due to lack of long-term results. At the same time, conventional valve replacement does not increase the risk of postoperative complications [5]. Taking into account the cusp morphology, we performed an aortic valve replacement due to the unpredictable outcome of the valve repair, which could jeopardize the overall result of the surgery [1]. Even though the patient did not reach the age criterion (65 years), we used the biological valve prosthesis in lieu of mechanical prosthesis to diminish the burden of antithrombotic therapy. The low incidence of the QAV emphasizes the importance of reporting such cases for a better understanding of the inherent characteristics of this rare anomaly. Evolving data will contribute to the development of QAV patient management strategies.

## Data Availability

The data presented in this study are available on request from the corresponding author. The data are not publicly available due to containing confidential doctor and patient information.
